# History of Nonalcoholic Fatty Liver Disease

**DOI:** 10.3390/ijms21165888

**Published:** 2020-08-16

**Authors:** Amedeo Lonardo, Simona Leoni, Khalid A. Alswat, Yasser Fouad

**Affiliations:** 1 Ospedale Civile di Baggiovara, UOC Medicina Metabolica, Dipartimento di Medicina Interna Generale, d’Urgenza e post Acuzie, Azienda Ospedaliero-Universitaria di Modena, Via Giardini 1135, 41125 Modena, Italy; 2 Internal Medicine Unit, Department of Digestive Diseases, S.Orsola-Malpighi Hospital, Via Massarenti 9, 40136 Bologna, Italy; simona.leoni@aosp.bo.it; 3 Liver Research Center, Department of Medicine, College of Medicine, King Saud University, Riyadh 11322, Saudi Arabia; kalswat@ksu.edu.sa; 4 Department of Gastroenterology, Hepatology and Endemic Medicine, Faculty of Medicine, Minia University, Minya 19111, Egypt; yasserfouad10@yahoo.com

**Keywords:** cryptogenic cirrhosis, genetics, guidelines, history of medicine, hepatocellular carcinoma, histopathology, insulin resistance, MAFLD, metabolic syndrome, molecular pathogenesis, NASH, pediatric NAFLD, steatosis.

## Abstract

Based on the assumption that characterizing the history of a disease will help in improving practice while offering a clue to research, this article aims at reviewing the history of nonalcoholic fatty liver disease (NAFLD) in adults and children. To this end, we address the history of NAFLD histopathology, which begins in 1980 with Ludwig’s seminal studies, although previous studies date back to the 19th century. Moreover, the principal milestones in the definition of genetic NAFLD are summarized. Next, a specific account is given of the evolution, over time, of our understanding of the association of NAFLD with metabolic syndrome, spanning from the outdated concept of *“NAFLD as a manifestation of the Metabolic Syndrome”*, to the more appropriate consideration that NAFLD has, with metabolic syndrome, a mutual and bi-directional relationship. In addition, we also report on the evolution from first intuitions to more recent studies, supporting NAFLD as an independent risk factor for cardiovascular disease. This association probably has deep roots, going back to ancient Middle Eastern cultures, wherein the liver had a significance similar to that which the heart holds in contemporary society. Conversely, the notions that NAFLD is a forerunner of hepatocellular carcinoma and extra-hepatic cancers is definitely more modern. Interestingly, guidelines issued by hepatological societies have lagged behind the identification of NAFLD by decades. A comparative analysis of these documents defines both shared attitudes (e.g., ultrasonography and lifestyle changes as the first approaches) and diverging key points (e.g., the threshold of alcohol consumption, screening methods, optimal non-invasive assessment of liver fibrosis and drug treatment options). Finally, the principal historical steps in the general, cellular and molecular pathogenesis of NAFLD are reviewed. We conclude that an in-depth understanding of the history of the disease permits us to better comprehend the disease itself, as well as to anticipate the lines of development of future NAFLD research.

## 1. Background

### 1.1. Definition

Formerly named nonalcoholic fatty liver disease (NAFLD), the spectrum of fatty liver disorders not resulting from alcohol abuse, viral, autoimmune, drug-induced and genetic etiologies, has recently been renamed metabolic (dysfunction) associated fatty liver disease (MAFLD) [1]. In agreement with a consistent line of opinions, this novel nomenclature correctly points out the “positive” determinants of the disease, namely the close association with metabolic disorders, rather than defining it for what it is not (i.e., nonalcoholic) [2]. Table 1 [1,3,4,5,6,7,8,9,10,11,12,13,14,15,16,17,18,19,20,21,22] lists some of the definitions that have either been proposed or used to designate NAFLD/MAFLD over time.

### 1.2. Burden

Obesity is an independent predictor of disease, accounting for the incremental changes in NAFLD over time in the USA [23]. However, NAFLD is not only common in USA and Europe, where it affects roughly one quarter of the general population [24]: in certain areas of the world, such as in South America, urban India and Sri Lanka, Israel and Turkey, prevalence rates of NAFLD range from 30% to 48% [25]. As a result of its epidemic distribution, NAFLD has become a major clinical and public health issue worldwide [26,27,28,29,30,31].

### 1.3. Aim

Given that the clue to future research is deeply eradicated in NAFLD history [32], and that understanding its historical developments over time will promote optimal practice, a group of researchers worked together to identify the principal steps in the study of NAFLD in adults and children. 

The history of NAFLD includes myriads of milestone advances and innumerable major breakthroughs which, collectively, would be virtually impossible to review. Therefore, four major areas of interest have been identified: histopathology; clinical correlates—natural course; guidelines, and general, cellular and molecular pathogenesis. Although these areas are mutually overlapping, this schematic partitioning serves to provide a more legible analysis.

## 2. History of NAFLD Histopathology

### 2.1. Before 1980

Addison was the first to describe fatty liver in 1836 [3]. Subsequently, for decades, pathologists pinpointed the similarities of liver histology changes seen in diabetic and morbidly obese individuals with those of alcoholics. In 1838, in autopsy specimens, the pathologist Rokitansky documented hepatic fat accumulation that might be causative of cirrhosis [33]. In 1884, Pepper described fatty infiltration of the liver in a diabetic patient [34]. In 1885, Bartholow reported a potential association between obesity and fatty liver [35]. In 1938, Connor described fatty liver infiltration that might led to the development of cirrhosis in diabetics. He reported on two cases of bleeding esophageal varices (one case was fatal owing to severe hemorrhage) in patients with diabetes and fatty liver. Perilobular fibrosis described in these patients was explained by both mechanical factors and tissue anoxia [4]. In 1958, Westwater and Feiner reported the histological findings of fatty infiltration of the liver in obese patients [36]. In 1962, Thaler added a further clinical and pathological description of the disease [36]. Since then, several reports in the 1950s–1970s pathologically documented the occurrence of fatty liver disease in obese and diabetic subjects [36].

### 2.2. 1980 and Beyond

In 1980, the term nonalcoholic steatohepatitis (NASH) was coined by Ludwig et al., to describe the progressive form of fatty liver disease histologically resembling alcoholic steatohepatitis though observed in patients who denied any alcohol abuse [7]. The majority of patients were obese women, and many were diabetic. The histopathological changes included lobular hepatitis, inflammatory infiltrates, Mallory bodies and focal necrosis with evidence of fibrosis in most specimens and cirrhosis in three patients [7]. In 1983, Moran *et al*., extended these findings to obese children in whom steatohepatitis presented with abnormal liver enzymes and non-specific symptoms [37]. Schaffner and Thaler were first to use the name “nonalcoholic fatty liver disease” in 1986 [9].

Over time, several histological scores for disease assessment have been developed and, currently, at least four main semi-quantitative scoring systems for the assessment of the histological features of NAFLD are available. The NAFLD activity score, comprised 14 histological features, 4 of which were evaluated semi-quantitatively: steatosis (0–3), lobular inflammation (0–2), hepatocellular ballooning (0–2), and fibrosis (0–4). Another nine features were recorded as present-or-absent. This score was developed by the NASH Clinical Research Network (NASH-CRN) [38]. The “Fatty Liver Inhibition of Progression (FLIP)” algorithm, which was developed by the FLIP consortium, is based on a scoring system (including steatosis, ballooning and lobular inflammation), the SAF score (steatosis, activity, fibrosis) [39]. The so called “Brunt” system score included ten histological variables to determine the inflammatory grading with a score for staging fibrosis [40]. Finally, the pediatric NAFLD histological score was based on the evaluation of steatosis, ballooning, portal inflammation and lobular inflammation [41].

There is general consensus that a constellation of histological features is required for the histopathological identification of adult NASH, including steatosis, ballooning, lobular inflammation and perisinusoidal fibrosis. In contrast, there is no universal agreement among liver pathologists regarding the essential criteria for the diagnosis of NASH. In addition, compared to other histological features, such as fibrosis, the histological diagnosis of NASH exhibits a large inter- and intra-observer variability and sampling error, which is reflected by the widely ranging prevalence of NASH, from 1.4% to 20% of liver biopsies [42]. This lack of reliability in the assessment of NASH may also affect NASH trials, by introducing patients who do not meet entry criteria, misclassifying fibrosis subgroups, and attenuating apparent treatment effects [43]. For instance, in the sole Phase 3 clinical trial for NAFLD to date that showed significant results, obeticholic acid failed to demonstrate a significant impact on NASH resolution, though it had a significant effect on fibrosis [44,45]. Future studies should identify reliable non-invasive tests for the prediction of NASH.

In this context, a panel of international experts from 22 countries across the globe recently proposed to abandon the simple and inaccurate dichotomous classification into ‘NASH’ versus ‘non-NASH’. These authors, aiming at improving the assessment of severity of disease, argue that the gamut of liver lesions should rather be assessed as a continuous and dynamic variable, such as is done in other diseases, therefore minimizing the negative implication of this conceptually wrong dichotomization [1,46].

Interestingly, steatosis may not persist during the progression of NAFLD, and rather may vanish in advanced cases of NAFLD-cirrhosis. This may lead to the blurring of the distinction between cryptogenic cirrhosis versus burned-out NAFLD-cirrhosis. Recently, various reports have demonstrated that features and the course of the two entities are different [47,48]. Unfortunately, this group of patients is usually excluded from clinical trials, as they lack the key criterion of “presence of steatosis”. The international consensus panel clarified this aspect by proposing that patients with cirrhosis, even in the absence of typical histological features of steatohepatitis, should be considered as MAFLD-related cirrhosis if they meet at least one of the following criteria: past or present evidence of metabolic dysregulation (according to MAFLD criteria), with either documentation of MAFLD in previous biopsy or steatosis by imaging techniques [17,46].

### 2.3. History of Genetic NAFLD 

NAFLD pathobiology has a high level of inheritability, and the genetic determinants of disease development and progression are increasingly recognized. Similar to other complex diseases, the genetic studies of NAFLD have passed through two major stages: the candidate gene approach first, followed by genome-wide association studies (GWAS) [49]. The former approach is driven by hypotheses based on the *a priori* knowledge of the biological functions regulated by candidate genes. Numerous variants of genes which can govern (therefore candidates) either susceptibility to or progression of NAFLD have been identified using this approach [50]. However, most of these studies were underpowered owing to small size, which has been reflected by the inconsistency of published reports.

The first GWAS in hepatology aimed at investigating the genetic basis of susceptibility to NAFLD dates back to 2008 [51]. Since then, hypothesis-free method-based discoveries, including GWAS, whole-genome and whole-exome sequencing have become the default methodology to determine genotype–phenotype associations. In these tests, correlations are performed between large numbers of single-nucleotide polymorphisms (SNPs), up to hundreds of thousands to over a million across the genome, and a single trait. This has led to an advancement in our understanding of the genetic underpinnings of NAFLD, with at least five variants in different genes having been robustly associated with the susceptibility to development and progression of NAFLD. These include: patatin-like phospholipase domain-containing protein 3 (PNPLA3), transmembrane 6 superfamily member 2 (TM6SF2), glucokinase regulator (GCKR), and hydroxysteroid 17β- dehydrogenase (HSD17B13) [51,52,53,54,55]. In addition, this approach helped in characterizing the genetic basis shared by NAFLD with other liver diseases as well as with other metabolic disorders, by identifying a role for variants in membrane bound O-acyltransferase domain-containing 7 (MBOAT7) [56,57,58], IFNL3/IFNL4 [59,60] and FNDC5 in NAFLD [61]. That said, it remains uncertain whether *“genetic NAFLD”* is perfectly equivalent to *“metabolic NAFLD”* as far as, for example, cardiovascular risk is concerned [62].

In the post-GWAS era, we are currently harvesting the benefits of the GWAS discoveries, including the incorporation of genetics in diagnostic and prognostic models [63,64], with an emerging role for polygenic scores [65,66]. In addition, genetic findings are well positioned to lead the path for modernization of the process of drug development, with recent evidence suggesting that a drug target with a genetic link has a double likelihood of success in clinical trials compared to other drugs that lack such a link [67,68]. Finally, the era of phenome-wide association study (PheWAS), moving from investigating a single phenotype to considering multiple phenotypes, is emerging [52].

## 3. History of Clinical Correlates and Natural Course of NAFLD 

### 3.1. From the Metabolic Syndrome to NAFLD

**Figure 1:**
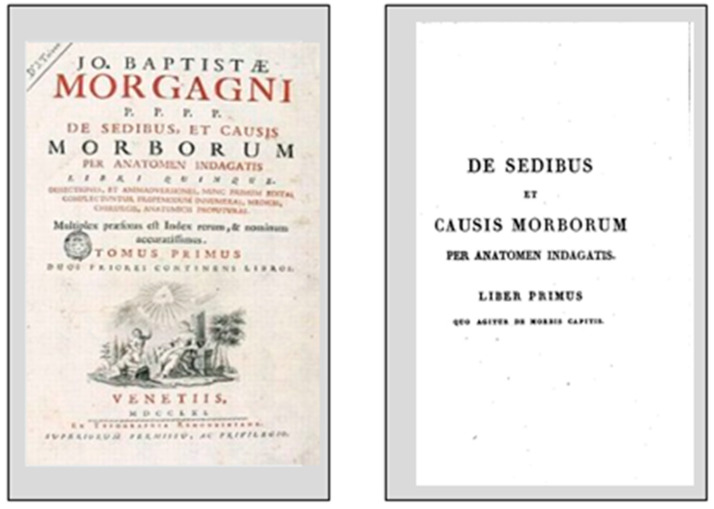
Joannes Baptista Morgagni’s ‘De Sedibus et Causis Morborum per Anatomen Indagata’.

The history of the metabolic syndrome is intriguing and complex. The first recognition of obesity and visceral adiposity as cardiovascular risk factors probably dates back to almost 2.400–260 years ago, respectively (Table 2 [69,70,71,72,73,74,75,76,77,78,79,80,81,82,83,84,85,86,87,88,89,90,91,92,93,94,95,96,97,98] and Figure 1 [73]).

In 1765, the Italian medical genius, JB Morgagni, lucidly identified the principal features of what we would now define as metabolic syndrome. He reported on the anatomical basis of “android obesity” and associated such pathological findings with hypertension, hyperuricemia, atherosclerosis and obstructive sleep apnea syndrome, long before the modern recognition of this syndrome [73]. This outstanding achievement descended from Morgagni’s mechanistic view of human physiology and pathology. He envisaged health as the result of the well-balanced functioning of the various organs. Conversely, any disease resulted from specific tissue damage, and this is still largely accepted in contemporary medical sciences [73].

However, most contributions belong to the 20th century. During the 1920s, Austrian, Swedish and Spanish authors reported on the association of arterial hypertension, diabetes, obesity, hyperuricemia, and vascular disease [99]. In the same decade, based on insurance data, it was observed that albuminuria/kidney disease, diabetes, cardio-circulatory disease, and high blood pressure clustered in overweight and obese individuals [100]. In 1939, Himsworth identified two different types of diabetes and established an association between insulin resistance and risk of type 2 diabetes [99]. In his seminal studies, conducted for almost 35 years, Vague and his group established a firm association between central distribution of body fat and unfavorable metabolic effects. However, it was not until the early 1980s that, owing to contributions by Kissebah and Bjorntorp, this concept became accepted [76]. 

The nomenclature of metabolic syndrome has been variable over time, including names such as hypertension–hyperglycaemia–hyperuricaemia syndrome, metabolic trisyndrome, plurimetabolic syndrome, syndrome of affluence, syndrome X, deadly quartet and insulin resistance syndrome [99]. More recent advances in operative definitions of metabolic syndrome are illustrated in Table 2 [69,70,71,72,73,74,75,76,77,78,79,80,81,82,83,84,85,86,87,88,89,90,91,92,93,94,95,96,97,98]. Studies highlighting the association of metabolic syndrome and NAFLD are shown in Table 3 [6,7,11,101,102,103,104,105,106,107,108,109,110,111,112,113,114,115,116,117,118,119,120,121,122]. Collectively, these studies were deemed to be consistent with the notion that NAFLD was “the hepatic manifestation of the Metabolic Syndrome”, which agrees with the popular motto that “fatty people have fatty livers”.

### 3.2. From NAFLD to the Metabolic Syndrome

A more recent line of research, however, has shown that the association of NAFLD with metabolic syndrome is mutual and bi-directional. For example, in the early 2000s, it became clear that surrogate indices of hepatic dysfunction predicted incident T2D and metabolic syndrome [123,124]. Bringing these epidemiological data further, it was possible to conduct theoretical as well as meta-analytic studies, showing that NAFLD was indeed a potential precursor of T2D and metabolic syndrome and that the stage of fibrosis was a strong determinant of such a risk [94,125,126].

### 3.3. NAFLD and Cardiovascular Risk

The liver was deemed to harbor life and soul in ancient Middle Eastern cultures, thus assuming a significance similar to that which the heart holds in our contemporary Western society [127,128]. On this historical background, a strong link between NAFLD and cardio-metabolic risk has recently been identified [129,130]. 

In 1995, Lonardo et al. hypothesized that NAFLD could be a clue that is useful in detecting cardiovascular disease [11]. In 2004 and 2005, Targher et al. were first to report that NAFLD was significantly associated with early carotid atherosclerosis in healthy men, and an increased risk of cardiovascular disease in patients with T2D, independent of classical risk factors, and that the occurrence of metabolic syndrome could account for this, to a partial extent [131,132]. Moreover, these authors also identified the stage of liver fibrosis as an independent predictor of carotid intima-media thickness, after the adjustment for potentially confounding factors such as metabolic syndrome [133]. Since 2005, several studies have confirmed that NAFLD is strongly associated not only with subclinical atherosclerosis [134], but also with major cardiovascular events. In 2016, Targher *et al.,* by meta-analyzing 16 unique, observational studies, enrolling a total of 34,043 adult individuals (36.3% had NAFLD), and evaluating nearly 2600 CVD events (>70% of which were CVD deaths) followed-up over a median period of 6.9 years, found that NAFLD patients, compared to controls without NAFLD, exhibited an increased risk of fatal and/or non-fatal CVD events. Moreover, those individuals who had “more severe” NAFLD, defined based on imaging techniques plus either elevated serum gamma-glutamyltransferase concentrations or high NAFLD fibrosis score or high 2-deoxy-2-[fluorine-18]fluoro-D-glucose uptake on positron emission tomography, or by biopsy-proven fibrosis stages, were also more likely to develop fatal and non-fatal events of cardiovascular disease [135]. Therefore, modern studies seemingly confirm the historical notion that the liver is involved in cardiocirculatory physiopathology [127].

### 3.4. NAFLD and Cancer

By the early 2000s, it had already become clear that NAFLD was associated with both hepatic and extra-hepatic cancers.

#### 3.4.1. Hepatocellular Carcinoma 

In 2002, two seminal studies reported on the risk of hepatocellular carcinoma (HCC) developing in the setting of NAFLD.

Bugianesi *et al.,* by retrospectively identifying 44 patients with HCC occurring in the setting of cryptogenic cirrhosis (CC) out of 641 cirrhosis-associated HCCs, observed that hypertriglyceridemia, diabetes, and normal aminotransferases were the risk factors independently associated with HCC arising in CC, suggesting that HCC may represent a late complication of NASH-cirrhosis [136].

Marrero *et al.,* by studying 105 consecutive cases of HCC, reported that either histological or clinical features associated with NAFLD were common among patients with CC; moreover, HCCs manifesting among patients with CC were larger at diagnosis given that they were less likely to have undergone HCC surveillance, and therefore these were less likely to be candidates for surgical or local ablative therapies [137].

Presently, the development of HCC in a subset of individuals is a definite feature of the natural course of NAFLD [129]. A meta-analytic review reported that, compared to other etiologies of liver disease, in non-cirrhotic subjects, those with NASH have a higher risk of HCC [138]. The risk factors for the development of HCC in those with NAFLD include genetics, lifestyle, liver-related and metabolic determinants [139,140]. 

#### 3.4.2. NAFLD and Extra-Hepatic Cancer

In their pioneer study, Sørensen *et al.,* by using the Danish National Registry of Patients, compared the Danish general population data of 7326 individuals who had received a hospital diagnosis of: alcoholic (ICD-8 _ 571.10), nonalcoholic (ICD-8 _571.11), or unspecified fatty liver (ICD-8 _ 571.19) at least once during the 16-year study period. Data have shown that patients with nonalcoholic/unspecified fatty liver had an increased risk of pancreatic cancer (standardized incidence ratio (SIR) 3.0; 95% CI, 1.3–5.8; vs. SIR 1.5; 95% CI, 0.7–3.0) and kidney cancer (SIR 2.7; 95% CI, 1.1–5.6) [141].

Presently, a variety of extra-hepatic cancers, including colorectal adenoma and carcinoma, are increasingly identified as a systemic manifestation of NAFLD [142,143]. Recent data suggest that NAFLD—more than obesity—is associated with an increased risk of extra-hepatic cancers, such as those of the gastrointestinal tract and uterus [144]. A meta-analytic review of observational studies of asymptomatic individuals submitted to colonoscopy, owing to screening purposes reported that NAFLD was independently associated with a mildly increased risk of incident and prevalent colorectal adenomas and cancer [145]. Various pathogenic mechanisms underlie the association of NAFLD with large bowel carcinogenesis, including sub-clinical systemic inflammation, IR, adipokines, bile acids and liver fibrosis [146,147]. 

## 4. History of Guidelines on NAFLD Issued by Scientific Societies 

Over time, scientific societies from different geographic areas have issued guidelines focusing on the criteria for diagnosis and management of NAFLD in adults, aimed at regulating clinical decision making. It is notable that a gap of decades separates the first clinico-pathological recognitions of NAFLD from recommendations issued by scientific societies. Probably, this mirrors the initial scarcity of evidence-based data to support strong recommendations. Distinctive features of the wide spectrum of NAFLD include expanding epidemiological trajectories, continuous progress in non-invasive diagnostic tools, as well as findings from basic research and clinical therapeutic trials of novel candidate drug regimens. All these concur in rendering publications and the updating of NAFLD guidelines a formidable multidisciplinary effort and an ongoing challenge for scientific hepatological societies. 

The first NAFLD guidelines were released by the Asian Pacific Association Study of the Liver (APASL) in 2007. This document was a summary of proposals by the Asian–Pacific Working Party for NAFLD, and was accompanied by reviews which summarized and annotated evidence and rationale supporting recommendations [148,149]. It was an informative effort directed at clinicians regarding a new globally expanding disease. Interestingly, these authors were able to find some common grounds in NAFLD management, although strong evidence was lacking at that time. This first document proposed by Asian scientific societies paved the way for the publication of clinical practice guidelines for NAFLD in Europe.

In 2010, the European Association for the Study of the Liver (EASL) issued a position statement that summarized the proceedings of the 2009 EASL Special Conference on NAFLD/NASH. This seminal article proposed expert opinion regarding different aspects of the clinical care of NAFLD patients [18].

In 2012, a NAFLD guidelines document was published as a collaborative effort from the three major American hepatological societies: American Association for the Study of Liver Diseases (AASLD), American College of Gastroenterology and American Gastroenterological Association [150]. These comprehensive guidelines included an extensive scientific literature search and followed the standard Grading of Recommendation Assessment, Development and Evaluation (GRADE) methodology [151]. 

To complete this first set of international NAFLD guidelines, in 2014, The World Gastroenterology Organization published a global NAFLD guidelines document, which is unique in following a resource-sensitive approach, i.e., a hierarchical set of diagnostic, therapeutic, and management options to deal with risk and disease, ranked by the resources available (Cascade) [152].

Between 2007 and 2014, either consensus statements or practice guidelines based on the recommendations of national societies were also issued. These include: the Italian Association for the Study of the Liver (AISF) in 2010 [153], the Chinese Association of The Study of Liver Disease in 2011 [154], the Korean Association for the Study of the Liver in 2013 [155], and the Japanese Society of Gastroenterology and the Japanese Society of Hepatology in 2015 [156]. 

The abundance and worldwide circulation of international and national guidelines witness that NAFLD is a global challenge. Concurrently, the high number and scientific standard of basic studies, clinical trials and informative review articles collectively attest that NAFLD remains an open and evolving paradigm for clinicians, needing further multidisciplinary approaches aimed at addressing the pathogenic heterogeneity, the multiple metabolic risk factors and the rapid epidemiological diffusion of disease. Major breakthroughs in our understanding of disease and evolving the medical practice fully justify a continuous updating of guidelines. Between 2016 and 2018, EASL, APASL and AASLD published the update of their first set of clinical recommendations. In particular, EASL worked in collaboration with the European Association for the Study of Diabetes and the European Association for the Study of Obesity, in developing the first multidisciplinary clinical practice guidelines on NAFLD in 2016 [157]. The 2016 EASL guidelines pay special attention to NAFLD screening in the population at risk. In 2018, APASL and AASLD published new consensus statements based on the most recent evidence [158,159,160]. 

Moreover, additional national societies either published novel or updated previous documents or guidelines. This is the case for NICE guidelines in 2016 [161], AISF in 2017 [129] and the Spanish Association for the Study of the Liver in 2018 [162]. Table 4 [18,129,149,150,152,153,154,155,156,157,158,159,160,161,162,163] is a synopsis of all the published guidelines.

The comparative analysis of NAFLD guidelines is an informative academic practice, identifying both shared and diverging key points [164]. The most updated of such comparative studies clearly highlights differences in the definition of alcohol threshold, choice of screening methods, identification of the best non-invasive tool for detecting liver fibrosis and the discussion of different pharmacological approaches [165]. There is general agreement regarding the notion that non-invasive tools such as NAFLD fibrosis score (NFS) and Fibrosis 4 score (FIB-4) and transient elastography or MRI should be used to detect patients with significant liver fibrosis. Moreover, scientific societies also agree that lifestyle changes, including healthy diet, habitual physical activity and weight loss are the mainstay of treatment. However, global management of NAFLD patients still varies across different geographical areas and different national healthcare systems [165,166].

It is expected that translation into clinical practice of those shared recommendations may result in improving homogeneity in NAFLD management, as well as improved outcomes in clinical trials. 

Although NAFLD has epidemic proportions in adults, children are not spared either [167]. Additionally, pediatric NAFLD has distinctive histological and pathogenic features, and is an ever escalating cause of chronic liver disease, with the potential of impacting health outcomes in adolescents and young adults [168]. This justifies the publication of NAFLD guidelines from pediatric scientific societies.

In 2017, practice guidelines on this topic were published by the North American Society for Pediatric Gastroenterology, Hepatology, Nutrition (NASPGHAN) and the update of AASLD guidelines on NAFLD included a pediatric section; this is a significant step towards providing diagnostic and therapeutic tools to optimize clinical care in children. The open questions in children are similar to those in adult populations: the identification of risk factors, screening strategies and screening tests, reference standard for the diagnosis, non-invasive biomarkers and imaging; lifestyle modifications as the first-line approach [160,169].

## 5. History of General, Cellular and Molecular Pathogenesis of NAFLD and NASH

Our understanding of the level of complexity of NAFLD pathogenesis has increased over time. While the earliest view had indicated the mechanistic development of steatohepatitis as a simple “two-hit” phenomenon, i.e., cell insults such as oxidative stress, lipid oxidation and inflammation superimposed on steatosis caused by IR [170], subsequent theories have clearly elucidated a more sophisticated level of complexity. In their seminal paper, Tilg and Moschen proposed that, irrespective of whether inflammation chronologically precedes or follows steatosis, many parallel hits of intestinal and/or adipose tissue origin, endoplasmic reticulum stress, (adipo)cytokines and innate immunity act in concert to regulate the distinctive features of NASH [171]. This “multiple hits hypothesis” continues to maintain its scientific credibility [172].

It would be difficult or even impossible to summarize here all the individual scientific contributions that, over time, have facilitated a more in-depth understanding of NAFLD and NASH pathogenesis. Excellent reviews may be consulted to this end [173,174,175]. That said, however, certain particularly innovative lines of research developed by distinguished groups of authors are acknowledged in Table 5.

## 6. Conclusions

Words of caution have recently been spent by eminent researchers regarding the risks inherent in a premature change of NAFLD nomenclature [216]. NAFLD and MAFLD are not exactly the same disease. A recent study conducted in 13,083 cases extracted from the NHANES III data has clearly documented this notion, by showing that MAFLD is more likely to capture those patients with hepatic steatosis, who exhibit a higher risk of disease progression [217]. Should these findings be confirmed, our understanding of relevant features of NAFLD, such as natural history and treatment response rates to lifestyle changes and experimental drug agents, may likely be in need of reassessment, if the MAFLD definition is accepted. 

We have tried to recapitulate the chief historical advancements in NAFLD, spanning histology, pathophysiology, pathogenesis and guidelines. We apologize to all those eminent authors who, inadvertently, are not mentioned here: their contributions have been acknowledged elsewhere [218]. Our review article has shown that there are some unsettled issues in the history of metabolic syndrome: why, for example, were ancient Indo-European physicians apparently aware of its existence (Table 2), whereas ancient Egyptian physicians were not ? [219,220]. Is this a clue to a healthy diet? [219]; or, does this result from North Africans being genetically spared from NAFLD and hence the MetS [24,221]?

An analysis of historical perspectives of disease has also revealed that many lines of current research, such as clinico-pathological correlations, personalized medicine, and sex differences are deeply eradicated in NAFLD history ( Table 2; Table 3). On these grounds, we emphasize that understanding the historical lines of research which have eventually conducted to present views may assist, particularly but not only, younger researchers, toward identifying the most appropriate research strategies to innovate, by giving significance to the past [32]. Stated otherwise, as summarized in this adage attributed to Johann Wolfgang Goethe, *“The history of a science is that science itself”.*

## Figures and Tables

**Table 1:** Names used in the past to designate NAFLD and MAFLD [1,3,4,5,6,7,8,9,10,11,12,13,14,15,16,17,18,19,20,21].

Year—Author [Ref]	Name	Comment
1845—Addison [3]	Fatty Liver	Thomas Addison, better known by the eponymic disease of cortisol deficiency, was first in reporting alcohol-induced liver histology changes.
1938—Connor [4]	Fatty infiltration of the liver	This author clearly pinpoints that steatosis, irrespective of whether owing to alcoholic etiology or due to diabetes, is a precursor of cirrhosis in animal studies as well as in humans.
1964—Dianzani [5]	Hepatic steatosis	This contribution, written in Italian, is the first of three highlighting the pathogenic mechanisms eventually conducive to the accumulation of intra-hepatic fat.
1979—Adler & Schaffner [6]	Fatty liver hepatitis and cirrhosis	In the obese, liver histology changes resemble those induced by alcohol and jejuno-ileal bypass suggesting a common denominator in these three conditions.
1980—Ludwig [7]	Nonalcoholic steatohepatitis (NASH)	In this seminal report of 20 patients whose liver biopsy specimens exhibited striking fatty and necro-inflammatory changes, Mallory bodies, fibrosis and cirrhosis, the name “NASH” is coined. The cohort featured a high prevalence of female sex; most patients were obese, many had T2D, gallstones and thyroid disease.
1985—Batman [8]	Diabetic hepatitis	Report of a nonalcoholic patient with a family history of both diabetes and chronic liver disease in whom liver histology resembling alcoholic hepatitis, asymptomatic chronic progressive hepatomegaly and mild alterations of liver tests preceded incident glucose intolerance by years.
1986—Schaffner & Thaler [9]	Nonalcoholic Fatty Liver Disease	This review article was first in using the name nonalcoholic fatty liver disease.
1988—Diehl, Goodman, Ishak [10]	Alcohol-like liver disease in the non-alcoholic	Liver histology features of alcoholic and nonalcoholic individuals were often indistinguishable based on histology alone suggesting that liver histology does not explain the clinical differences between these individuals and raising the possibility that either nutritional or hormonal factors account for alcohol-like histological changes in both conditions.
1995—Lonardo [11]	Bright liver syndrome	This was meant to be an umbrella definition grouping together (hence “syndrome”) the conditions observed in individuals with a “bright liver echopattern” at ultrasonography. The associations with gallstones and atherosclerosis are highlighted.
1999—Mendler [12]	IRHIO	Probably NAFLD, such as is found in a population with a high prevalence of hereditary hemochromatosis
2002—Neuschwander-Tetri and Caldwell, on behalf of AASLD [13]	MESH	The authors summarize the presentations and discussions at an AASLD-sponsored Single Topic Conference on fatty liver disorders held in September, 2002.
2002—Dixon [14]	MSSH	The disease entity of NASH includes many putative factors. These authors propose to enucleate a condition which is clearly related to the MetS, hence the name MSSH.
2002—Farrell [15]	NASH	The author discusses the prevalence, importance and risk factors of NAFLD in the Asia-Pacific region. A proposed classification of weight by body mass index for Asians is devised in addition to a practical approach to the diagnosis of NAFLD.
2004—Brunt [16]	NASH	The author discusses the prevalence studies and the pathophysiology of NAFLD including the challenges of pediatric NASH and NASH-related cirrhosis.
2005—Loria, Lonardo and Carulli [17]	Metabolic (fatty) liver disorders	The reasons why NAFLD should be renamed are extensively discussed. It is proposed that a positive criterion being introduced in the name would create significant benefits. It is also highlighted that fatty changes disappear when cirrhosis develops explaining the superior importance of “metabolic/insulin resistance” over “fatty” in the qualification of this syndromic liver disorder.
2009—Ratziu [18]	Metabolic fatty liver disease	This position paper strongly argues for a change in NAFLD nomenclature by dropping the ‘‘negative” definition (‘‘nonalcoholic”) and recognizing the key role of IR.
2009—Brunt [19]	Metabolic fatty liver disease	The definitions of NAFLD/NASH remain based on the “non-association” with alcoholic etiology rather than with the recognition of those truly associated conditions.
2011—Balmer and Dufour [20]	MAFLD	Based on the recognition that AFLD and NAFLD share the same liver histology and often also metabolic alterations, the authors believe that MAFLD might describe both patient populations more accurately while depicting the key pathophysiological features.
2017—Bellentani and Tiribelli [21]	NAFLD and NASH could be collectively named MAFL. MAFL associated with liver injury may be designed as MASH, or DCH.	The authors list the several designations to identify NAFLD, such as BASH, CASH, DASH or GASH. They suggest progressing from a negative to a positive definition.
2019—Eslam, Sanyal & George [22]	MAFLD	Proposal of more accurate nomenclature of disease. This study lays the foundation for the work of an International panel of experts published the following year.
2020—Eslam, Sanyal & George on behalf of the International Consensus Panel [1]	MAFLD	An International panel of experts from 22 countries proposes a novel definition of disease which is based on hepatic steatosis, associated with one out of three criteria: overweight/obesity, T2D, metabolic derangement.

AASLD—American Association for the study of Liver Diseases; AFLD—alcoholic fatty liver disease; BASH—alcoholic and non-alcoholic steatohepatitis; CASH—chemotherapy-associated Steatohepatitis; DASH—drug-associated steatohepatitis; DCH—dysmetabolic chronic hepatitis; GASH—genetic-associated steatohepatitis; IR—insulin resistance; IRHIO—Insulin-resistance-associated hepatic iron overload; MAFL—metabolic-associated fatty liver; MAFLD—metabolic (dysfunction) associated fatty liver disease; MASH—metabolic-associated steatohepatitis; MESH—metabolic steatohepatitis; MSSH—metabolic syndrome steatohepatitis; T2D—type 2 diabetes.

**Table 2:** Principal advances in the history of Metabolic Syndrome [69,70,71,72,73,74,75,76,77,78,79,80,81,82,83,84,85,86,87,88,89,90,91,92,93,94,95,96,97,98].

Year—Author [Ref]	Findings	Comment
Around 600 BC—Sushruta [69]	It is believed that ancient Ayurvedic medicine was probably first in envisaging a nexus linking excess weight and diabetes. Sushruta described diabetes (madhumeha or “honey-like urine”), as featuring the passage of large amounts of sweet-tasting urine, primarily affecting obese, sedentary individuals predisposed to developing angina (“hritshoola”). Sushruta was the first physician recorded to have prescribed exercise to cure diabetes and obesity. Exercise is described as “moderate in nature or to an intensity that will cause laboured breathing”	The reasons why features of the MetS were described by Indo-European as opposed to North-African clinicians is not completely clear.
c. 460–c. 370 BC—Hippocrates [70,71]	Hippocrates had clear ideas regarding the fact that obesity was associated with sexual dysfunction in either sex. Moreover, he had observed cases of sudden deaths among obese people (*Those who are constitutionally very fat are more apt to die quickly than those who are thin.)* and advised lifestytle changes to his patients. However, an aphorism says *The more severely diet is restricted, the sooner will the rebellious patient break the rule, will overeat and later suffer both for the doctor’s and his own mistake*. A more active lifestyle was also among Hippocrates’ precepts: *Walking is man’s best medicine.*	It would seem that Hippocrates had already understood that obesity poses multiple health risks and that lifestyle changes, i.e., dieting (without excessive restrictions) and exercising are useful in contrasting these.
Avicenna—981 [69]	The very obese are at risk of a fatal rupture of a blood vessel. They are vulnerable to stroke, hemiplegia and palpitation.	A lucid analysis of cardiovascular risk associated with obesity.
1765—Morgagni JB [72]	A 74-year-old lady with severe obesity and an android aspect died owing to stroke. At autopsy intrabdominal and intramediastinal cavities were filled with a large amount of fat. A flabby, obese sedentary gentleman engaged in literature studies, and indulgent towards opulent meals had a short, thick “bull-like” neck”. At the age of 40 he started passing pink-orange colored bladder stones, with urine. At the age of 61 he started complaining of headaches and sleepiness. When he was 63 he developed peripheral edema, aphasia, right side hemiplegia and eventually died. Necropsy identified bladder stones; reddish lungs; enlarged heart and severe calcific atherosclerosis of the carotid and vertebral arteries.	Although laboratory tests and imaging techniques were not available in the 18th century, the anatomo-clinical reports written by JB Morgagni are deemed to be the very first description of visceral obesity and related complications in either sex [73].
1924—Joslin [74]	*“Diabetes is 15 times as common among* *adults and 20 times as common among the fat”*	Elliott Joslin was the first US doctor specialized in diabetes care.
1939—Himsworth [74]	*“On the whole the sensitive diabetics tend to be younger and thin and to have a normal blood pressure and normal arteries, and as a rule their disease is of sudden and severe onset.* *The insensitive diabetics, on the other hand, tend to be elderly and obese and to have hypertension and arteriosclerosis, and in these patients the onset is insidious”*	This Author identified two different types of diabetes, of which “insensitive diabetes” is what we now call T2D.
1947—Vague [75]	This pioneering paper was published in French at the end of World War II. Vague reports that relative hyperanabolism, the basis of obesity and sexual differentiation work together to produce android, gynoid or mixed obesity.	This seminal work by Vague, published in French, is deemed to probably have suffered from a limited availability of resources owing to World War II [76]. However Vague continued his studies for many years and, in 1956, he reported on the association of android obesity with the risk of developing diabetes, hypertension, gout and atherosclerosis [77].
1967—Avogaro [78]	A report of 6 mildly obese patients with stable non-ketonuric diabetes-induced hyperlipidemia. Treatment with a low-calorie, low- carbohydrate diet, induced weight loss, was effective in normalizing/markedly decreasing fasting blood glucose and serum triglyceride values.	A lucid analysis of the close association linking carbohydrate intake with metabolic derangements.
1969—Feldman [79]	This study shows that central body fat distribution is associated with the development of T2D	Although confirmative of the findings previously reported by Vague, this paper failed to gain scientific awareness.
1982—Kissebah [80]	9 non-obese apparently healthy women and 25 obese women, were evaluated. Plasma glucose, insulin levels during oral glucose loading, triglycerides and diabetes were associated with obesity predominantly affecting the upper body segment. Upper body obesity was comprised of large adipocytes, while adiposity in the lower body was formed of normal-sized cells. In both types of obesity, the size of abdominal adipocytes was significantly associated with postprandial plasma glucose and insulin levels. Thigh adipocytes were resistant to epinephrine-stimulated lipolysis.	In women, impaired glucose disposal, hyperinsulinemia and hypertriglyceridemia occur as a result of the specific morphology and metabolic attitudes of adipocytes associated with upper body obesity.
1983—Krotkiewski [81]	This study showed with stepwise multiple regression analyses that, in both women and men, the complications of obesity were linked to waist/hip circumference.	This is the first of a series of studies conducted by these authors. A subsequent study published in 1984 established the concept that, in middle aged men, the distribution of fat deposits may better predict CVD and death than the degree of adiposity [82].
1987—Fujioka [83]	The association of intra-abdominal adipose tissue (evaluated with CT scan) and disorders of glucose and lipid metabolism was evaluated in 46 obese individuals. The V/S ratio was significantly correlated with the level of plasma glucose AUC of 75 g OGTT, with triglyceridemia and total cholesterolemia.	The accumulation of intra-abdominal fat predisposes to impaired glucose tolerance and dyslipidemia in obese individuals.
1988—Reaven [84]	This lecture lucidly describes the chain of pathophysiological events which occur in most of the patients with either IGT or T2D and in a quarter of non-obese glucose normo-tolerant individuals. Hyperinsulinemia may effectively prevent frank decompensation of glucose homeostasis at the price of developing HTN, hyperglycemia, dyslipidemia, and CAD.	This Banting Lecture raises the possibility that resistance to insulin-stimulated glucose uptake and hyperinsulinemia are involved in the development and progression of T2D, HTN, and CAD
1989—Kaplan [85]	The evidence that upper-body obesity, which usually occurs as a result of calorie excess in the presence of androgens, predisposes to hypertension, diabetes, and hypertriglyceridemia even in the absence of significant overall obesity mediated by hyperinsulinemia.	There is a need to identify and prevent upper-body obesity. Whenever this fails, therapies should be provided that would control the “deadly quartet” without worsening hyperinsulinemia.
1991—Ferrannini [86]	Among 2930 subjects from the general population, the prevalence of obesity, T2D, IGT, HTN, hypertriglyceridemia, and hypercholesterolemia alone, two by two or in association was evaluated. The large differences in prevalence between isolated and mixed forms suggest important overlapping among the six components of the MetS. In their isolated forms, each condition was characterized by hyperinsulinaemia which is evidence for IR. Fasting and post-glucose hyperinsulinaemia was associated with higher BMI, WHR, fasting and post-glucose glycaemia, systolic and diastolic BP, serum triglycerides and total cholesterol levels and lower HDL-cholesterol concentrations.	IR, glucose intolerance, HTN, body fat mass and distribution, and serum lipids are a network of mutually interrelated functions; each and all of the six disorders increase the risk of CAD.
1999—WHO Alberti and Zimmet [87]	Proceedings of a meeting held in London in 1996 under the sponsorship of Bayer, Novo and The Institute for Diabetes Discovery. This document also incorporated subsequent comments from the Experts.	This document was first to include insulin resistance as a diagnostic criterion of the MetS.
1999—Balkau & Charles [88]	The EGIR proposed that 3 out of 5 clinical criteria were sufficient to define the MetS.	Diagnostic criteria proposed by EGIR were IR and ≥ 2 criteria among central obesity, high triglycerides or low HDL, HTN, and fasting glucose ≥ 6.1 mmol/L.
2001—NCEP [89]	Updated clinical guidelines for cholesterol testing and intensive cholesterol-lowering treatment in clinical practice. An evidence-based and extensively referenced document report which provides the scientific foundations for the recommendations contained in the executive summary.	These guidelines are meant to inform rather than replace clinical judgment.
2002—Ford [90]	In this analysis of data on 8814 adult men and women from the 3rd NHANES (1988–1994). The age-adjusted prevalence rate of the MetS was 23.7%, similar in men and women but varied based on ethnicity. Using 2000 census data, about 47 million US residents have the MetS.	Based on 2000 census data, about 47 million US residents were estimated to have the MetS carrying major implications for health care.
2004—Grundy [91]	The scientific foundations underlying the definition of MetS was considered from several perspectives spanning from metabolic components and pathogenesis to criteria for diagnosis, clinical outcomes and therapeutic interventions.	The primary outcomes of MetS are CVD and T2D. T2D will further contribute to increasing CVD risk. ATP III criteria are practical in identifying patients at increased risk for CVD. Irrespective of the diagnostic criteria used, lifestyle changes, notably including weight loss, represent a first-line therapeutic approach for MetS.
2005—Grundy [92]	MetS defines a constellation of endogenous risk factors which predispose to ASCVD and T2D. MetS is multi-factorial and exhibits major inter-individual, inter-racial and inter-ethnic variability.	In the USA, the MetS is strongly associated with abdominal obesity. Lifestyle changes are the first-line interventions and drug therapies for individual risk factors may be indicated whenever lifestyle changes fail.
2005—Kahn [93]	Concerns are raised regarding diagnostic criteria; rationale for using thresholds in biological parameters; the importance of including diabetes in the definition; uncertainty as to IR as the unifying etiology; absence of clear grounds for including/excluding other CVD risk factors; variable value in assessing the risk of CVD; failure of the MetS to identify CVD risk more accurately than its individual components; management of the MetS overlapping with that of each of its constitutive components; the added value of diagnosing the syndrome is uncertain.	The principal value in identifying the MetS is based on the notion that the individual components of the MetS tend to cluster in the same individuals and each of these often foreruns the incidence of additional components over time. Along with the risk of progressing to target organ failure (e.g., cirrhosis) and of the development of some cancer types (e.g., HCC) makes MetS a relevant diagnosis for practicing clinicians and a global major public health problem [94]
2005—Reaven [95]	While the concept of IR provides a pathophysiologic framework bringing together a number of seemingly unrelated biological phenomena, the MetS is a pragmatic approach aimed at making a diagnosis to initiate lifestyle changes and decreasing CVD risk.	The diagnosis of the MetS will not promote our pathophysiologic understanding or clinical utility: deciding that individuals do not have the MetS owing to their failure to satisfy 3 out of 5 arbitrary criteria may withhold important therapeutic decisions.
2009—Alberti [96]	The MetS defines HTN, atherogenic dyslipidemia, hyperglycemia, and central obesity —which are risk factors for CVD and T2D - and tend to cluster more often than due to chance alone. Various diagnostic criteria have been proposed by different organizations over time which chiefly differ regarding the measurement of central obesity.	This statement tries to unify existing criteria. It was concluded that there should not be an obligatory component, but that waist measurement would be a useful screening tool. 3 out of 5 abnormal findings would qualify an individual for the MetS. National/regional cut-off values for WC can be used.
2010—Simmons [97]	Conclusions of a WHO Expert consultation evaluating the utility of the concept itself of MetS ‘as related to epidemiology, physiopathology, clinical aspects and public health.	The notion of MetS focuses on complex multifactorial health problems. Therefore, it is useful as an educational concept while its clinical value as a diagnostic or management tool is quite limited. Perspectives for future research are also discussed
2016—Lopes [98]	In this excellent review of the history of the MetS, these authors call it VAS.	The definition of VAS is well taken in as much as it highlights the key anatomical basis underlying metabolic derangements which had astutely been identified by Morgagni.

ASCVD—atherosclerotic cardiovascular disease; BP—blood pressure; CAD—coronary artery disease; CVD—cardiovascular disease; EGIR—european group for the study of insulin resistance; HCC—hepatocellular carcinoma; HTN—arterial hypertension; MetS—metabolic syndrome; NCEP/ATP III—National Cholesterol Education Program/Adult Treatment Panel III; NHANES—National Health and Nutrition Examination Survey; T2D—type 2 diabetes; VAS—visceral adiposity syndrome; V/S ratio—visceral fat to subcutaneous fat ratio; WC—waist circumference; WHO—World Health Organization; WHR—waist to hip ratio.

**Table 3:** Principal advances in the history of the association of NAFLD with the Metabolic Syndrome.

Year—Author [Ref]	Method	Findings	Comment
1935—Zelman [101]	Review of experimental pathology and clinical science.	*“Although the increased incidence in human obesity of gallbladder disease and diabetes mellitus^4^ has long been known, and although these conditions may lead independently to liver disease, there does not appear to be in the literature a consideration of the existence of liver damage in obesity per se. The majority of obese persons show a significant decrease in carbohydrate tolerance, and this impairment has been related to the duration rather than to the degree of obesity. Similar decreases in carbohydrate tolerance are observed in the experimental obesity of hypothalamic injury and in hereditarily obese mice.”*	A clear allusion to what we would now call “MAFLD”. This study also reports on the risk of developing a specific form of progressive NASH secondary to injuries of the hypothalamic-hypophyseal axis.
1970—Beringer and Thaler [102]	465 liver biopsies performed in diabetics.	Being overweight, rather than diabetes duration or metabolic control, was associated with the severity of hepatic steatosis. Among various forms of treatment, only treatment with insulin was significantly associated with the degree of steatosis.	Most of these patients had maturity-onset diabetes associated with obesity.
1977—Haller [103]	The Dresden study addressed the most important CVR factors.	Obesity 8.2%, hyperlipoproteinemia 7.4%, hyperuricemia 3.8%, T2D 2.0%, hypertension 17.2% and smoking 30.3% were the most common CVR factors.	“MetS” is defined as the concurrence of obesity, T2D, hyperlipoproteinemia, hyperuricemia, and hepatic steatosis. Haller recognizes this MetS as being associated with increased risk of artheriosclerosis owing to increased blood viscosity and procoagulant state.
1979—Itoh [104]	A report of five cases.	Five nonalcoholic diabetic women over 50 years of age who had obesity and hyperglycemia, were found to have clinically and histologically proven micronodular cirrhosis.	The histological findings differed from cirrhosis following hepatitis and developed owing to centrilobular necrosis.
1979—Adler and Schaffner [6]	Criteria for inclusion: obese subjects (≥50% overweight for height based on Insurance Company standards) referred owing to either hepatomegaly or abnormal liver tests. Criteria for exclusion: excessive alcohol consumption, drug abuse, acute or chronic liver disease; HBsAg; AMA; biopsy-proven chronic hepatitis. abnormal imaging findings regarding gallbladder or bile duct; impaired renal function; abnormal results of routine blood counts; disease of the digestive tract; infection or cardiac decompensation. Based on liver histology, patients were classified into one of the following categories: Group I. Fatty liver; Group II. Fatty hepatitis; Group III. Fatty fibrosis and Group IV. Fatty cirrhosis.	The age range was 18 to 69 years, average 46 years. Their weights ranged from 150% to 300% of ideal weight for height). Female to male ratio was 22:7. A family history of diabetes or CAD was found in 34%; a previous Cholecystectomy for gallstones was found in 28%. 14% had diabetes under oral antidiabetic agents and 65% were taking cardiovascular drugs. The degree of liver damage was inversely related to the proportion of lipoprotein abnormalities (71% in groups I and II) vs. 36% in groups III and IV (*p* < 0.01).	In obese individuals presenting with either clinical or laboratory evidence of liver disease, all the histological spectrum of alcoholic hepatitis can be observed. Prevalence of the female sex, a high incidence of gallstones, HTN, T2D and hyperlipo-proteinemia (predominantly type IV of Frederickson’s classification) were the most prominent clinical features found in this series of obese patients.
1980—Ludwig [7]	Findings in 20 patients with NASH are reported.	Liver biopsy findings exhibited striking fatty changes with lobular hepatitis, focal necroses with mixed inflammatory infiltrates, Mallory bodies and fibrosis. Three had cirrhosis. The disease was more common in obese women most of whom also had T2D and gallstones.	This seminal study was first in associating the novel name NASH with its clinico-pathological correlates.
1989—Lee [105]	A retrospective analysis yielded 49 cases of NASH out of 543 liver biopsies diagnosed as alcoholic hepatitis. Follow-up information after an average duration of 3.8 years was available for 39 patients	NASH tends to be a mild condition with the potential to progress to cirrhosis in some patients owing to unknown mechanisms.	In this pioneering study devoted to identifying the natural history of disease female sex, obesity and diabetes were prominent features of disease.
1990—Powell [106]	Forty-two NASH patients were followed for a median of 4.5 yrs (range = 1.5 to 21.5 yrs). All were obese except for two who had lipodystrophy. 35/42 were women, 26/32 had hyperlipidemia and 15/32 hyperglycemia.	NASH is a low-grade and slowly progressing chronic hepatitis resembling alcoholic liver disease which may, however, ultimately result in cirrhosis.	Decompensated diabetes and rapid weight loss preceded the onset of NASH. Severity of obesity, hyperlipidemia or hyperglycemia was not associated with the histological type/severity of disease.
1994—Bacon [107]	A series of 33 patients with NASH is analyzed.	All patients were HCV-Ab-negative. 58% were men, and 39% had pathologically increased liver fibrosis, 5 of whom had micronodular cirrhosis. NASH was also found in men without any obvious metabolic risk factors. However, those 13 cases with more advanced fibrosing disease, were predominantly obese women, with either T2D or IFG and hyperlipidemia. No patient had hemochromatosis although 58% displayed abnormal values of transferrin saturation and ferritin.	The NASH spectrum should be expanded as compared to Ludwig’s initial description [7].
1995—Lonardo [11]	A series of 339 patients submitted to ultrasonography scanning owing to clinical indications is evaluated. A minority of individuals were either HCV-Ab positive and all drank < 20 g alcohol daily, 21.5% had a “bright liver”.	Among those with a bright liver echopattern there was a prevalence of men. Overweight, arterial hypertension, gallstones, (previously undiagnosed) impaired glucose disposal, raised apoB and Lp(a) serum levels and clinical manifest atherosclerotic vascular disease were common.	A bright liver echopattern is often associated with extrahepatic multisystem involvement and could be a clue to identifying metabolic and cardiovascular diseases.
1999—Cortez-Pinto [108]	Body composition (with bioimpedance spectroscopy) and energy expenditure (with indirect calorimetry) were assessed in 10 patients with biopsy-proven steatosis, 20 with NASH and 8 healthy controls.	The prevalence of features of the Mets in NAFLD was as follows: obesity and dyslipidaemia 80% each; HTN 50%; T2D 33%; impaired glucose metabolism 69%. Hyperinsulinemia and hyperleptinemia were common. Insulin and leptin were mutually associated and correlated with BMI, fat mass and body fat percentage.	NAFLD is strongly associated with features of the MetS. Such an association is mediated by concurrent IR and leptin resistance.
1999—Lonardo [109]	A Medline research of the literature covering the years 1990–1998 and cross references was conducted.	Fatty liver typically affects middle aged men with features of the MetS such as obesity, altered glucose disposal, hyperlipidemia and HTN	The similarities of fatty liver with the MetS span epidemiology, anthropometry, metabolism, clinical features and experimental models.
1999—Marchesini [110]	Anthropometric and metabolic variables were evaluated in 46 patients with normo-glucose tolerant NAFLD [defined by chronically raised serum transaminases, compatible ultrasound scanning and exclusion of competing etiologies of liver disease]; and compared to 92 age- and sex- matched healthy controls.	NAFLD cases exhibited (fasting and glucose-induced) hyperinsulinemia, IR, asymptomatic postload hypoglycemia, and hypertriglyceridemia. The independent predictors of NAFLD were IR, fasting TG serum concentrations, 180-min blood serum glucose level and average insulin concentration following oral glucose. The exclusion of overweight and obese subjects did not alter these findings.	Normo-glycemic NAFLD, obesity and T2D belong to the same spectrum of disease which is associated with hyperinsulinemia, IR and hypertriglyceridemia. Either life-style changes or insulin-sensitizing agents may break the association of hyperinsulinemia, IR, hyperTG, thereby halting the progression of liver steatosis.
1999—Marceau [111]	551 (112 men) morbidly obese individuals submitted to bariatric surgery were evaluated.	Steatosis was found in 86%, fibrosis in 74%, mild inflammation/steatohepatitis in 24%, and unexpected cirrhosis in 2. The risk of steatosis was 2.6 times greater in men than in women. Per each addition of 1 of the 4 components of the MetS, the risk of steatosis increased exponentially. Fibrosis was correlated with steatosis and the presence of either diabetes or IGT carried a 7-fold increased risk of fibrosis. T2D, steatosis, and age were all significant indicators of cirrhosis.	The MetS is strongly associated with steatosis, fibrosis, and cirrhosis via IGT.
2002—Lonardo [112]	60 patients with NAFLD and 60 age and sex-matched controls were analyzed.	Patients exhibited hypertriglyceridemia, hyperuricemia, hyperisulinemia and obesity more often than controls. No iron storage was found among those who underwent liver biopsy.	Only fasting insulin and serum uric acid rather than indices of iron metabolism were independent predictors of NAFLD
2004—Donati [113]	55 patients who had arterial hypertension but were non-obese, non-diabetic, not drinkers of large amounts of alcohol and had normal liver enzymes were compared to 55 age- and sex- matched healthy controls.	Among patients with HTN, NAFLD was more prevalent and these patients were also more insulin resistant and had higher BMIs than controls. At LRA IR (OR 1.66, 95% CI 1.03–2.52) and BMI (OR 1.22, 95% CI 1.00–1.49) were independently associated with NAFLD; moreover, IR was predicted by ALT (*p* = 0.002), HTN (*p* = 0.029), and BMI (*p* = 0.048).	IR and higher body weight account for the higher prevalence of NAFLD among non-obese hypertensive patients with normal liver enzymes.
2005—Suzuki [114]	529 individuals who drank < 14 g alcohol/wk and were HBV and HCV negative were selected and a sub-cohort of 287 IR-free related features subjects were identified. Otherwise unexplained raised transaminases were used as a surrogate index for NAFLD. High transaminases, together with weight gain of > 2 kg and IR-related features in the sub-cohort were sought for up to 5 yrs.	Weight gain preceded low LDL cholesterol, hypertriglyceridemia, hypertransaminasemia, HTN, and glucose intolerance	This study clearly identifies chronological ordering of the individual features of the MetS in the development of surrogate indices of NAFLD.
2007—Kotronen [115]	Features of the MetS, other features of IR (serum insulin, C-peptide), visceral and sc fat (with MRI), LFC (with MRS) and transaminases were evaluated in 271 non-diabetic subjects.	LFC was 4-fold higher in subjects with than without the MetS independent of age, sex, and BMI. All features of the MetS were correlated with LFC. LFC was significantly correlated with transaminases, fasting serum insulin and C-peptide.	Excess of LFC is associated with the development of the MetS irrespective of BMI.
2007—Chitturi and Farrell [116]	Editorial commenting on two studies published in the same issue of the journal.	Studies indicating that NAFLD is a pre-diabetic condition are reviewed. Data useful to answering the question as to whether liver usltrasonography can be used to identify patients at risk for metabolic disease are critically evaluated.	The Authors propose that LFC may become a “barometer of metabolic health”. This brilliantly metaphoric definition still retains all its diagnostic and therapeutic utility.
2008—Musso [117]	197 unselected non-obese non-diabetic subjects were evaluated cross-sectionally. HOMA-IR > 2, oxidative stress (nitrotyrosine), soluble adhesion molecules (ICAM-1, VACM-1 and E-selectin) and circulating adipokines (TNF-α, leptin, adiponectin and resistin) were correlated to ATP III criteria for the diagnosis of the MetS and to NAFLD	IR was more accurately predicted by NAFLD than ATP III criteria. Accuracy in diagnosing IR was improved by adding NAFLD to ATP III criteria. Moreover, at LRA, NAFLD was an independent predictor of HOMA-IR, nitrotyrosine, and soluble adhesion molecules at LRA; the presence of NAFLD entailed more severe oxidative stress and endothelial dysfunction, independent of MetS-related confounding factors in subjects with IR.	In non-obese non-diabetic subjects NAFLD is more closely associated with IR, oxidative stress and endothelial dysfunction than MetS identified with ATP III criteria.Therefore, in this patient population, NAFLD may help in identifying subjects at increased cardiometabolic risk.
2010—Vanni [118]	Narrative review	Emphasis is placed on data suggesting that hyperinsulinemia, rather than causing, probably results from pre-existing NAFLD.	One of the first published papers focusing on the mutual and bi-directional realtionship linking NAFLD with the MetS.
2012—Hamaguchi [119]	Cross-sectional survey of 11,714 apparently healthy Japanese adult men and women submitted to a medical health checkup. NAFLD was identified with ultrasonography after excluding competing causes of liver disease. Revised criteria of the NCEPT III were used to identify MetS.	Although NAFLD is deemed to be the hepatic manifestation of MetS, the prevalence of MetS in NAFLD was low in either sex. When participants were defined as “positive at screening for NAFLD”, those who satisfied at least one criterion of MetS, had indeed NAFLD with a good sensitivity (84.8% in men and 86.6% in women).	In epidemiological studies NAFLD can effectively be identified by modified criteria of MetS.
2015—Zhang [120]	Based on a large-scale health check-up in a Chinese population, two bidirectional longitudinal subcohorts were identified and followed from 2005 to 2011: Subcohort A [i.e., from NAFLD to MetS, *n* = 8426 included those participants (with or without NAFLD at baseline) to follow-up the incidence of MetS], and Subcohort B [i.e., from MetS to NAFLD, *n* = 16,110 included those participants (with or without MetS at baseline) to follow-up the incidence of NAFLD].Generalized estimating equation analyses were conducted to assess the role of NAFLD as a potential causal factor for MetS and of MetS as a risk factor for incident NAFLD. A BN with 5 simplification strategies was used in order to infer reciprocal causality.	NAFLD was a potential causal factor for MetS and MetS was also a factor for NAFLD (2.55, 2.23 to 2.92). The total effect of NAFLD on MetS was 2.49%, while it was 19.92% for MetS on NAFLD. The total impact of NAFLD on MetS components was different, with dyslipidemia having the greatest effect, followed by obesity, diabetes and HTN. As for the effect of MetS components on NAFLD, obesity had the greatest effect, followed by T2D, dyslipidemia and HTN. The most important causal pathway from NAFLD to MetS was that NAFLD led to elevated GGT, then to MetS components, while the dominant causal pathway from MetS to NAFLD began with dyslipidaemia.	A reciprocal causality links NAFLD and MetS. The impact of MetS on NAFLD is significantly greater than that of NAFLD on MetS.
2014—Yki-Järvinen [121]	Narrative Review	Definitions of NAFLD and MetS are analyzed. The role of NAFLD as a predictor of cardio-metabolic disorders is extensively examined. Acquired and genetic causes of NAFLD and MetS as well as the pathomechanistic basis underlying the association are reviewed.	NAFLD not associated with PNPLA3 polymorphisms is closely reminiscent of MetS in terms of etiologies and outcomes. In these patients, LFC is an accurate barometer of metabolic health. Groups of individuals at a high risk for NAFLD include otherwise unexplained deep venous thromboembolism and gallstone disease. NAFLD predicts T2D better than MetS. Therefore, the diagnosis of NAFLD must invariably prompt the search for the MetS and its individual components. Conversely, the diagnosis of NASH should be pursued among all patients with the MetS. Lifestyle changes e.g., dietary restrictions (particularly of simple sugars) and increased physical activity must be proposed to both those with NASH and those with the MetS.
2017—Ma [122]	Prospective study of 1051 participants (mean age 45 ± 6 years, 46% women) followed for approximately 6 yrs.	Two analyses were conducted. Baseline liver fat (per each SD increase) was associated with increased odds of incident hypertension and T2D. In parallel, compared to individuals free of these conditions, subjects who at the baseline had HTN, hypertriglyceridemia, IFG, impaired fasting glucose or T2D had a higher risk of developing incident FL. In both analyses, findings persisted following further adjustments for measures of adiposity.	This study supports a bi-directional relationship associating FL and CVD risk factors in the 3rd generation cohort of the Framingham Heart Study.

ALT—alanine transaminase; AMA—anti-mitochondrial antibody; AST—aspartate aminotransferase; ATP III—adult treatment panel III; BMI—body mass index; BN—bayesian network; CVR—cardiovascular risk; FL—fatty liver; HBsAg—Hepatitis B surface Antigen; HOMA—homeostasis model assessment; HBV—hepatitis B virus; HCV—hepatitis C virus; HTN—arterial hypertension; ICAM—intracellular adhesion molecule-1; IFG—impaired fasting glucose; IR—insulin resistance; LFC—liver fat content; LRA—logistic regression analysis; MetS—metabolic syndrome; MRI—magnetic resonance imaging; MRS—proton magnetic resonance spectroscopy; NASH—nonalcoholic steatohepatitis; NCEPT—national cholesterol education program adult treatment panel; SC—subcutaneous; SD—standard deviation; TG—triglycerides; T2D—type 2 diabetes; VACM-1 vascular cell adhesion molecule-1.

**Table 4:** Guidelines, ordered by year of publication, published in English by different national and international Scientific Societies on NAFLD in adult population.

Year—Author [Ref]	Scientific Societies	Title
2007—Chitturi [149]	APASL	NAFLD in the Asia-Pacific region: definitions and overview of proposed guidelines.
2010—Ratziu [18]	EASL	A position statement on NAFLD/NASH based on the EASL 2009 special conference.
2010—Loria [153]	AISF	Practice guidelines for the diagnosis and management of NAFLD. A decalogue from the AISF Expert Committee.
2011—Fan [154]	Chinese Association of The Study of Liver Disease	Guidelines for the diagnosis and management of nonalcoholic fatty liver disease: update 2010
2012—Chalasani [150]	AASLD-ACG-AGA	The diagnosis and management of non-alcoholic fatty liver disease: practice guideline by the AASLD, ACG and AGA.
2013—Lee [155]	KASL	KASL clinical practice guidelines: Management of nonalcoholic fatty liver disease.
2014—LaBrecque [152]	WGO	World Gastroenterology Organisation global guidelines: Nonalcoholic fatty liver disease and non-alcoholic steatohepatitis.
2015—Watanabe [156]	Japanese Society of Gastroenterology and The Japanese Society of Hepatology	Evidence-based clinical practice guidelines for nonalcoholic fatty liver disease/nonalcoholic steatohepatitis.
2016—[Marchesini] [157]	EASL-EASD-EASO	EASL-EASD-EASO Clinical Practice Guidelines for the management of non-alcoholic fatty liver disease.
2016—no authors listed [161]	NICE	Non-Alcoholic Fatty Liver Disease: Assessment and Management.
2017—Lonardo [129]	AISF	AISF position paper on NAFLD: Updates and future directions.
2018—Chalasani [160]	AASLD	The diagnosis and management of nonalcoholic fatty liver disease: Practice guidance from the AASLD.
2018—Wong [158]	Asia-Pacific Working Party on Non-alcoholic Fatty Liver Disease	Asia-Pacific Working Party on NAFLD guidelines 2017-Part 1: Definition, risk factors and assessment.
2018—Chitturi [159]	Asia-Pacific Working Party on NAFLD	The Asia-Pacific Working Party on Non-alcoholic Fatty Liver Disease guidelines 2017-Part 2: Management and special groups.
2018—Aller [162]	Spanish Association for the Study of the Liver	Consensus document. Management of NAFLD.
2019—Alswat [163]	Saudi Association for the Study of Liver Diseases and Transplantation	Position statement on the diagnosis and management of NAFLD.

AASLD—American Association for the study of Liver Disease; ACG—American College of Gastroenterology; AGA—American Gastroenterological Association; AISF—Italian Association for the Study of the Liver; APASL—Asian Pacific Association Study of the Liver; EASD—European Association for the Study of Diabetes; EASL—European Association for the Study of the Liver; EASO—European Association for the Study of Obesity; KASL—Korean Association for the Study of the Liver; NAFLD—nonalcoholic fatty liver disease; NASH—nonalcoholic steatohepatitis; NICE—National Institute for Health and Care Excellence (UK); WGO—World Gastroenterology Organization.

**Table 5:** Principal advancements in cellular and molecular pathogenesis of NAFLD and NASH.

Years—Authors [Ref]	Topic	Comment
1999–2009—Caldwell; Leclerq; Robertson; Sanyal; Parardis; Crespo; Marra; Caldwell [176,177,178,179,180,181,182,183]	Oxidative stress and molecular fibrogenesis	A seminal line of research investigating the interconnections between metabolic dysregulation, hepatocyte mitochondrial abnormalities, TNF-alpha and fibrogenesis. These studies identify molecular pathways to be targeted for effective NASH drug treatment.
2004–2005—Targher, Kaser; Pagano; Vuppalanchi; Bugianesi; Targher [184,185,186,187,188,189]	Adiponectin	Adiponectin is an adipokine with anti-inflammatory and anti-steatotic properties. Hypoadiponectinemia is a feature of NAFLD. Adiponectin may also be associated with specific features of liver histology in NASH.
2005 Younossi; [190,191]	Genomic/proteomic analysis to obesity-related NAFLD	The molecular pathogenesis of disease was investigated by evaluating those differentially expressed genes/gene products in patients with NASH. These are related to lipid metabolism and extracellular matrix remodeling. Moreover, genes involved in liver regeneration, apoptosis, and the detoxification process were also differentially expressed.
2005 – Baffy [192]	UCP2	UCP2 is a widely distributed fatty acid-responsive carrier protein of the mitochondrial inner membrane. It is substantially increased in fatty liver where it may play a role at multiple steps including lipid metabolism, mitochondrial bioenergetics, oxidative stress, apoptosis, and carcinogenesis.
2007 – 2011 Puri; Puri; Fon Tacer; Bell [193,194,195,196]	Lipid deregulation and peroxidation are key features of NASH	Multiple alterations in the hepatic lipid composition characterize NAFLD. Moreover, the progression of NASH is associated with impaired PUFA metabolism and non-enzymatic oxidation. Perturbed lipid and lipoprotein metabolism accompanied by chronic inflammation is the central molecular pathway for the development of MetS-related diseases, including atherosclerosis, CVD and NAFLD. Hepatic lipid peroxidation is increased in children with NAFLD.
2005–2009—Feldstein and Gores; Malhi and Gores; Gentile and Pagliassotti; Farrell [197,198,199,200]	Apoptosis and molecular mechanisms of lipotoxicity and ER stress.	Apoptosis is a specific form of cell death that plays a key role in the pathogenesis of NAFLD. The subcellular and molecular mechanisms involved in triggering hepatocyte apoptosis are pinpointed. FFAs directly activate the proapoptotic protein Bax, in a c-jun N-terminal kinase-dependent manner. Moreover, FFAs activate the lysosomal pathway of cell death and regulate death receptor gene expression. Saturated fatty acids may represent the “second hit” hastening the development of NASH.
2008—Kallwitz; George [201,202]	PPARs	PPARs are nuclear hormone receptors. These, by acting as intracellular sensors for a variety of lipophilic molecules including cholesterol metabolites, and FFAs, play key roles in regulating energy homeostasis, steatogenesis, inflammation and IR.
2008–2011 Gronbaek; Kumashiro [203,204]	Molecular mechanisms linking NAFLD with IR and T2D.	These studies focus on the role of dietary fat, adipocytokines and the SREBP-1c in the association of IR and steatosis. Importantly, it is shown that IR in humans is best predicted by DAG content in hepatic lipid droplets supporting the notion that NAFLD-associated IR is caused by an increase in hepatic DAG content, which results in the activation of PKCε.
2009—Baffy [205]	Role of Kuppffer cells	TLRs (especially TLR4) activate Kuppffer cells following the recognition of danger signals. In NAFLD, this process may be perturbed at multiple steps owing to altered sinusoid microcirculation and impaired hepatocellular clearance of exogenous and endogenous danger signals; deranged lipid homeostasis; perturbed adipokine secretion and increased production of ROS.
2009—Miele [206]	Intestinal permeability	First evidence in humans that NAFLD is associated with increased gut permeability caused by disruption of intercellular tight junctions in the intestine, and leading to an increased prevalence of SIBO in these patients therefore contributing to the pathogenesis of hepatic fat deposition.
2009—Syn [207]	Hh-mediated EMT	Based on cell cultures and mouse NAFLD models it is concluded that Hh-mediated EMT in ductular cells contributes to the pathogenesis of cirrhosis in NAFLD.
2010—Cheung [208]	miRNA	Progress in miRNA research allows the molecular characterization of events that limit protein expression, which is key in NAFLD development and progression.
2011–2013—Van Rooyen [209,210,211]	Free cholesterol	This milestone research has consistently shown that SREBP-2 connects IR, hepatic cholesterol, and inflammation in NASH; that the cause of NASH in an experimental obese, diabetic mouse model is the accumulation of hepatic free cholesterol; and that cholesterol lowering with a combination of ezetimibe/atorvastatin reverses hepatic free cholesterol which dampens JNK activation, ALT release, hepatocyte apoptosis, and inflammatory changes, collectively leading to the reversal of fibrosing NASH in obese, diabetic mice with MetS.
2013—Pirola [212]	Epigenetic modification of liver mitochondrial DNA	Hepatic methylation and transcriptional activity of the mitochondrially encoded NADH dehydrogenase 6 are associated with the severity of NAFLD histology.
2015—Kasumov [213]	Ceramides are key mediators of cardio-metabolic risk in NAFLD	In LDLR(-/-) mice, a western diet-induced model of NAFLD and atherosclerosis caused hepatic oxidative stress, inflammation, apoptosis, increased hepatic long-chain ceramides associated with apoptosis (C16 and C18) and decreased very-long-chain ceramide (C24) involved in insulin signaling. The plasma ratio of ApoB/ApoA1 (proteins of VLDL/LDL and HDL) was doubled due to increased ApoB production. Myriocin decreased lipogenesis, ApoB production and increased HDL turnover. These changes translated into reduced hepatic and plasma ceramides and sphingomyelin, and decreased atherosclerosis, hepatic steatosis, fibrosis, and apoptosis.
2018—Kutlu [214]	Cancerogenesis	Molecular signaling pathways involved in NASH-derived HCC include genetic or epigenetic modifications and alterations in metabolic, immunologic and endocrine pathways that are closely associated with inflammation, liver injury and fibrosis in NASH.
2020—Hernández [215]	EVs are emerging as key players in the molecular pathogenesis of NAFLD	EVs contain a variety of bioactive molecules (e.g., proteins, lipids, coding and non-coding RNAs and mitochondrial DNA) that exert a key role in cell-to-cell communication via the secretion by different cell types. Stressed/damaged hepatocytes release large quantities of EVs that contribute to the progression of liver disease by affecting inflammation, fibrogenesis and angiogenesis.

ApoB—apolipoprotein B; ALT—alanine transaminase; CVD—cardiovascular disease; EMT—epithelial-mesenchimal transition; ER—endoplasmic reticulum stress; EVs—extracellular vesicles; FFAs—free fatty acids; HCC—hepatocellular carcinoma; HDL—high-density lipoprotein; Hh—hedgehog; IR—insulin resistance; JNK—c-Jun N-terminal kinase; LDLR—low-density lipoprotein receptor; MetS—metabolic syndrome; miRNA—microRNA; NASH—nonalcoholic steatohepatitis; PKCε—protein kinase C ε; PPARs—peroxisome proliferators-activated receptors; PUFA—polyunsaturated fatty acid; ROS—reactive oxygen species; SIBO—small intestine bacterial overgrowth; SREBP-1c—sterol regulatory element-binding protein-1c; TLRs—toll-like receptors; T2D—type 2 diabetes; UCP 2—uncoupling protein-2.
